# COVID-19, Cation Dysmetabolism, Sialic Acid, CD147, ACE2, Viroporins, Hepcidin and Ferroptosis: A Possible Unifying Hypothesis

**DOI:** 10.12688/f1000research.108667.2

**Published:** 2022-02-28

**Authors:** Attilio Cavezzi, Roberto Menicagli, Emidio Troiani, Salvatore Corrao

**Affiliations:** 1 Eurocenter Venalinfa, San Benedetto del Tronto, AP, 63074, Italy; 2 University of Milan, Milan, Italy; 3 Cardiology Unit, Social Security Institute, State Hospital, Cailungo, 47893, San Marino; 4 Department of Clinical Medicine, Internal Medicine Division,, ARNAS Civico Di Cristina Benfratelli Hospital Trust, Palermo, Italy

**Keywords:** ferroptosis; cations; sialic acid; iron; ferritin; calcium; viroporins; voltage-gated calcium channels; cell membrane; CD147; ACE2; hepcidin; red blood cells; hemoglobin; mitochondria

## Abstract

**Background: **iron and calcium dysmetabolism, with hyperferritinemia, hypoferremia, hypocalcemia and anemia have been documented in the majority of COVID-19 patients at later/worse stages. Furthermore, complementary to ACE2, both sialic acid (SA) molecules and CD147 proved relevant host receptors for SARS-CoV-2 entry, which explains the viral attack to multiple types of cells, including erythrocytes, endothelium and neural tissue. Several authors advocated that cell ferroptosis may be the core and final cell degenerative mechanism.

**Methods**: a literature research was performed in several scientific search engines, such as PubMed Central, Cochrane Library, Chemical Abstract Service. More than 500 articles were retrieved until mid-December 2021, to highlight the available evidence about the investigated issues.

**Results**: based on COVID-19 literature data, we have highlighted a few pathophysiological mechanisms, associated with virus-based cation dysmetabolism, multi-organ attack, mitochondria degeneration and ferroptosis. Our suggested elucidated pathological sequence is: a) spike protein subunit S1 docking with sialylated membrane glycoproteins/receptors (ACE2, CD147), and S2 subunit fusion with the lipid layer; b) cell membrane morpho-functional changes due to the consequent electro-chemical variations and viroporin action, which induce an altered ion channel function and intracellular cation accumulation; c) additional intracellular iron concentration due to a deregulated hepcidin-ferroportin axis, with higher hepcidin levels. Viral invasion may also affect erythrocytes/erythroid precursors, endothelial cells and macrophages, through SA and CD147 receptors, with relative hemoglobin and iron/calcium dysmetabolism. AB0 blood group, hemochromatosis, or environmental elements may represent possible factors which affect individual susceptibility to COVID-19.

**Conclusions**: our literature analysis confirms the combined role of SA molecules, ACE2, CD147, viroporins and hepcidin in determining the cation dysmetabolism and final ferroptosis in the cells infected by SARS-CoV-2. The altered ion channels and electrochemical gradients of the cell membrane have a pivotal role in the virus entry and cell dysmetabolism, with subsequent multi-organ immune-inflammatory degeneration and erythrocyte/hemoglobin alterations.

## 1. Introduction

The present narrative review aims at providing a summary of the available evidence about a few peculiar aspects of COVID-19 pathophysiology, regarding: a) alterations of cation (iron and calcium mainly) metabolism, b) the role of sialic acid (SA) molecules, CD147, ACE2 and of the ion-channeling components, such as voltage-gated calcium channels (VGCC) and viroporins, in the morpho-functional chemical/electric alterations of the cell membrane caused by the viral attack, c) the contribution of hepcidin, red blood cell (RBC) and hemoglobin to the cation dysmetabolism, d) the consequent intracellular degeneration, ultimately resulting in both a mitochondrial degeneration and the ferroptosis process.

Our literature search addressed the published articles related to COVID-19, which reported data and elaborations concerning the pathophysiological elements described above.

We performed literature research retrieving and reviewing pertinent articles and documents from the following web-based scientific search engines: MEDLINE, PubMed Central, Cochrane Library, Google Scholar, ChemRxiv, MedRxiv, BioRxiv, Preprints, ResearchGate, Chemical Abstract Service, Genetic Home References and Human Metabolome Database. The words COVID-19 and/or SARS-CoV-2 were combined with the following headings and keywords: ferroptosis, iron, ferritin, transferrin, hepcidin, ferroportin, calcium, potassium, sodium, magnesium, hemoglobin, heme, hematology, erythrocyte, red blood cells (RBC), erythroblast, RDW, LDH, hemochromatosis, chelation, mitochondria, lactate, CD147, sialic acid (SA), VGCC, ion channels, viroporins.

We investigated the biomedical literature since January 2020 through mid-December 2021, and moreover we reviewed the earlier reference articles where basic concepts and data were available for the main topics covered in this review ( *e.g.*, ferroptosis, iron, ferritin, SA, CD147, viroporins).

Nearly 500 articles were collected and reviewed, in order to extrapolate pertinent data and speculations concerning those clinical and instrumental (mainly laboratory biochemistry and computational biology) findings which pertain the investigated key-words.

## 2. Literature-based background

COVID-19 seems to be an extremely complex, variable systemic disease which has spread as pandemic worldwide, generating dramatic health and socio-economic problems. The responsible coronavirus, SARS-CoV-2, shares some of the pathomechanisms with the previous SARS-CoV-1 virus, but COVID-19 features peculiar clinical and instrumental findings.

A series of pathophysiology mechanisms have been described in these patients, mostly based on virus-induced immune-inflammatory pathways and on lung pathological consequences. In fact, a high body of evidence has been published about the multi-organ viral attack, which is not solely based on the original immune degeneration induced by the virus. Beyond the conventional pro-inflammatory pathways, other basic and possible pathomechanisms have been proposed in the early literature about COVID-19, ^1^ based on the preliminary scientific data.

Several authors have highlighted that iron dysmetabolism (and probable blood components denaturation), together with cell membrane morpho-functional deregulation, represent specific early biochemical phenomena in this infection. ^1^^–^^6^

Hyperferritinemia, low serum iron and low total iron binding capacity (TIBC) have been definitely linked to COVID-19 severity, and these elements have been considered relevant prognostic factors. ^1^^,^^7^^–^^14^ Similarly, a series of possible treatments aimed at controlling iron dysmetabolism have been proposed. ^15^^–^^19^ Together with these biochemical changes, a number of RBC alterations have been described, both in terms of hemoglobin decrease/denaturation, and in terms of RBC altered morphology/functionality. ^1^^,^^20^^–^^23^

Another well-documented cation dysmetabolism, especially in later stages is represented by lower serum calcium levels, which is equally a proven negative prognostic factor in COVID-19 patients ^24^^–^^32^; additionally, early autopsies also highlighted a higher representation of calcium deposits in many tissues. ^33^

Regulation of transmembrane pumps and calcium influx/efflux is generally of great importance in many viral infections. ^34^ Furthermore, past literature data has proven how excessive intracellular calcium does contribute to mitochondria dysfunction and to ferroptosis degeneration. ^35^

In view of the clinical relevance of excessive intracellular calcium, the use of calcium channel blockers has been repeatedly suggested in the patients affected by COVID-19. ^36^^–^^41^

In confirmation of the possible value of these drugs, a recently published retrospective cohort study among 4569 hospitalized hypertensive patients with COVID-19 showed a dramatic reduction of mortality in patients treated with calcium channel blockers (risk ratio [RR]: 0.32, 95% confidence interval [CI]: 0.13-0.76, P = 0.0058). ^42^

Decreased calcium levels in blood, in absence of a documented hypercalciuria, indicate a detrimental increased concentration of this cation inside the cells. The movement of this mineral across cellular membranes is highly regulated through the VGCC, which have an important role also in multiple pathological clinical conditions involving muscular, neural, cardiac and endothelial cells, just to name a few. ^43^^–^^48^

A possible effect of SARS-CoV-2-mediated altered transmembrane cation passage in the endothelium may regard the electromotive force oscillations and ultimately the endothelial contractility. In fact, two preliminary studies have highlighted an increased arterial stiffness in these patients, in comparison to age-matched healthy subjects. ^49^^,^^50^

Interestingly, patients with later stages of COVID-19 often show altered serum concentration of other cations, such as sodium, potassium and magnesium. ^51^^–^^54^

Pathophysiology of this disease and, more specifically, cation dysmetabolism is linked to a series of viral interactions with human cell membrane receptors.

Beyond the ascertained role of ACE2, several authors have reported the determinant role of another peculiar receptor, namely CD147, in the multi-organ viral attack. ^1^^,^^55^^–^^59^

CD147, also termed Basigin or EMPRIM, is regarded as a relevant SARS-CoV-2 entry port with a specific role in a few tissues. ^1^^,^^55^^,^^60^ However, it is worth mentioning that a minority of literature publications denies a direct role of this receptor in the whole viral process. ^61^

In fact, CD147 is represented in several human tissues and for example it is widely localized on the erythrocyte membrane (nearly 2000-3000 receptors per cell are described, ^62^ erythroblasts, endothelial cells, brain *etc.* ^1^; as it is supposed that SARS-CoV-2 may affect also RBC, the putative role of CD147 is sustainable. ^63^

A few publications focused on CD147 in COVID-19; in fact, this diffusely distributed cell membrane receptor is a well-known mediator in several diseases, where it exhibits a pleiotropic function. ^64^^–^^66^

Cluster of Differentiation 147, or CD147, is a highly glycosylated transmembrane glycoprotein belonging to the immunoglobulin superfamily, compelling for the Ok blood group system. The immunoglobulin superfamily comprehends proteins with at least one Ig domain and is of fundamental importance in intercellular communication. ^67^

This transmembrane protein is dedicated to recognizing molecules inside the cell (cis-recognition), and extracellularly (trans-recognition). Various isoforms of human CD147 are produced by differential splicing and differences in transcription initiation sites. Isoform-1 has three Ig domains, located in the retina. Isoform-2, the most common, has two Ig domains. ^68^ Intriguingly, human platelets also express CD147, and a few authors observed a SARS-CoV-2 spike protein-dependent platelet activation and aggregation, which is in fact CD147-mediated. ^57^

CD147 was also found to contribute as a chaperone of plasma membrane transport of monocarboxylate transporters, hence a few authors postulated the possibility that CD147 might participate in ACE2-virus interaction, by a re-localization to the same compartment of the two receptors. ^58^

Furthermore, the well-known role of CD147 in inflammatory processes, together with its upregulation during hypoxic states, ^1^^,^^69^ may contribute to the deregulated inflammatory pathways of this disease in several organs. More specifically, it was found that the viral interaction with CD147 may lead to some documented detrimental viral effects on endothelium ^70^^–^^72^ and on RBC and their precursors. ^1^^,^^21^^,^^63^^,^^73^^,^^74^

In addition, it has been observed that CD147 expression levels correlate with SARS-CoV-2 infection extent, vascular damage and an increased expression of vascular endothelial growth factor and thrombosis. ^75^

Likewise, cardiovascular tissues and endothelial cells express putative genes for SARS-CoV-2 infection, including the ones regarding ACE2 and Basigin/CD147. However, ACE2 decreases with age in some tissues, whereas basigin/CD147 increases with age in endothelial cells, suggesting that the expression of this specific receptor in the vasculature might explain the heightened risk for severe vascular diseases with age. ^71^^,^^76^ Interestingly, in most COVID-19 autopsy studies a diffused micro- and macro-vascular thrombosis was shown. ^77^

Beyond the importance of ACE2 and CD147 receptors, several authors have pointed out how SA role should be re-assessed and emphasized in COVID-19. Early in May 2020, the determinant function of SA in SARS-CoV-2 transmembrane attack was highlighted. ^78^ More recently, several authors have similarly remarked a series of SA-based interaction pathways between the virus and the host cell. ^59^^,^^79^^–^^85^

Sialic acids are ubiquitous structural polysaccharides that are typically found attached to the N and O terminal positions of the cell membrane glycoproteins. Their structure is unique in the class of glycans: these alpha-keto acids have nine carbon atoms and this helps them to play a critical role in several intrinsic and extrinsic cell interactions. The SA family includes many derivatives of neuraminic acid and currently more than 50 structural variants for SA have been found in nature ^86^; this molecule has also been implicated in various pathophysiological processes such as oncogenesis and microbial pathogenesis. ^87^ In fact, several viruses attack host cells by binding with particular sialylated glycans, which become cell adhesion molecules, to mediate cell entry. ^88^

Of great importance, it has been recognized that SA may be expressed on the outer layer of cell membrane as molecules located on specific wall glycoproteins, but also on the surface of ACE-2 and CD147 receptors. ^89^ Sialo-glycoconjugates expressed on cell surfaces serve also as ligands or receptors for specific intrinsic or extrinsic SA lectins.

Host cell receptors undergo evolutionary alterations to avoid rapidly emerging pathogens while maintaining critical endogenous function. Similarly, most viruses have evolved to express enzymes that can cleave the interactions with these SA receptors, helping to release them from the infected host cells. These sialidases act as decoy receptors, which bind to virions, preventing their access to epithelial cells as well. ^87^

The presence or absence of an appropriate host SA receptor is a major determinant of the viral host tropism, including the specific host tissue and cell types that viruses can infect. Host receptors are therefore one of the keys to understanding susceptibility to a particular virus and to determining the body systems that are likely to be infected and the type of clinical outcome. ^85^^,^^90^^,^^91^

Blood AB0 group, as well as blood viscosity and erythrocyte shape, are strictly dependent upon the RBC wall negative electrical charge, which is induced mainly by the SA molecules; hence RBC morphology strictly relates to the configuration and density of these negatively charged molecules on their membrane. ^92^^–^^97^

With reference to the RBC changes induced by pathogens, it was also demonstrated that several viral glycoproteins may interact with SA on the erythrocytes of various species, resulting in agglutination. ^87^^,^^98^

The expression and distribution of these SA-based receptors differ according to their location within the body, cell type, and their functional role. Humans predominantly express SA α2,6-Gal receptors in the ciliated and non-ciliated epithelium of the respiratory tract, but also in blood, gut and neural system. ^99^ Additionally, age-dependent differences in the distribution of SA receptors have been reported in human subjects. ^59^

Beyond the cell membrane negative charge, also osmolarity and pH of the intra/extracellular space are significantly influenced by SA concentration and disposition. Hence, SA molecules are being considered of central importance in the SARS-CoV-2/cell docking and consequent infection. ^78^^,^^85^^,^^91^^,^^100^

Past research showed that electrical and pH changes due to SA deregulation or its interaction with pathogens, proved to interfere with cell membrane morpho-functional condition, for example resulting in spherical RBC shape and consequently in altered blood viscosity. ^78^^,^^101^^,^^102^

There is growing evidence that sialylated compounds present in the cellular glycocalyx may serve as an important factor in the mechanism of COVID-19 infection. A few studies have specifically focused on the glycosylation process of the two subunits of the spike protein, S1 (which facilitates the attachment to the host cell receptor through the receptor binding domain, RBD) and S2 (which mediates the fusion of viral to human cell membrane). ^103^^,^^104^

In a very recent paper, unexpected changes in the glycosylation of the S1 RBD have been shown, which can explain the crucial role of SA molecules in viral binding to ACE2, CD147 and to the glycoproteins of the host cell membrane. ^105^

Equally, in a computational biology paper it has been reported that the average estimated binding affinity is much higher for SA molecules (both free or on ACE2) docking with the furins and the cleaved spike protein subunit S1, than for any other receptor/compound on the cell wall. Interestingly, they found that binding of negatively charged SA molecules of host glycoproteins to the SARS-CoV-2 S protein will target the furins more thoroughly than the ACE2s, which may increase the efficiency of furin cleavage. ^106^

In general, coronaviruses bind SAs on the RBC and on the epithelium of upper respiratory tract through the hemagglutinin glycoproteins. The higher or lower concentration of these molecules on the various cell membranes, which may also depend upon a genetic constitution, is supposed to play a role in different diseases as well as in COVID-19. ^6^^,^^107^^,^^108^

Blood AB0 group determination is based also upon SA distribution on RBC wall glycoproteins and there is some contrasting evidence about the possibility that AB0 group may have a prognostic role in patients affected by COVID-19. ^109^

Interestingly, the blood group A has been found to have a more preferential SARS-CoV-2/RBD docking with the erythrocytes and with the cells of the respiratory epithelium. ^110^ Similarly, it has been proposed to link both the possible 0 group protection, and the A group higher susceptibility, to the different glycosylated part of the erythrocyte membrane (hence to the SA component). ^111^

However, some opposing data are reported in other studies where it is shown that SA molecules content does not depend on AB0 blood type. ^112^ Overall, several factors influence AB0 group determination, thus much more evidence is likely needed before drawing sound conclusions about this topic.

As a result of the described dysfunctional metabolic pathways in RBC, erythroblasts and hemoglobin, an alteration of iron metabolism and possibly also of the oxygen-transport function is expected. In fact, low levels of hemoglobin have been found in worse clinical stages, ^7^^,^^113^^–^^115^ while, conversely, higher hemoglobin levels at the time of the hospital admittance were associated to worse prognosis. ^116^^,^^117^

Furthermore, a number of publications have documented a clear increase in the figures of a few biomarkers which express dysfunctionality of hemoglobin and RBC, such as bilirubin, lactate dehydrogenase (LDH) and red cell distribution width (RDW). ^1^^,^^117^^–^^120^

Based on the evidence above, a multiple pathway-hypothesis has been published in the early 2021 to explain erythrocyte and hemoglobin denaturation in these cases. ^121^

Additionally, considering the possible autoimmune hemolysis (and piastrinopenia) consequent to the viral interaction with RBC receptors (namely CD147 and sialylated membrane glycoproteins), ^122^^–^^125^ the possibility of free circulating heme has been taken in consideration with reference to the hypothetical pathomechanisms of COVID-19. ^1^^,^^126^^–^^132^

More recently, an indirect demonstration of the SARS-CoV-2-induced hemolysis has been reported through liquid chromatography, with a documented increase of protoporphyrin IX in blood of COVID-19 patients. ^133^

Overall, scientific research has early focused on the possible role of hemoglobin morpho-functional alterations in this disease, as reported above, though a homogenous and direct evidence about hemoglobin dysregulation in COVID-19 has not been reached; similarly, literature reports contrasting papers about the related hypoxia and about the altered hemoglobin dissociation-curves. ^4^^,^^134^^–^^141^

At the same time, also the critical debate about hypoxemic hypoxia and about the high-altitude pulmonary edema (HAPE)-like condition in COVID-19 patients has attracted some scientific interest. ^142^^–^^144^ In fact, the dysfunctionality of RBC and hemoglobin has been proposed as primary cause of hypoxemic hypoxia, in presence of normal lung functionality during the early phase of COVID-19; the subsequent lung parenchyma deterioration in the late phase, with pneumolysis and interstitial pneumonia, would finally contribute to the ARDS phenomenon and to the deteriorated hypoxic state. ^1^^,^^145^^–^^148^

Iron and hemoglobin metabolism are strictly related; if the latter presents some denaturation, it is expected that there is some repercussion on iron biochemical pathways. However, this is a reminder here that iron physiology is strictly regulated by the axis hepcidin/ferroportin. Hepcidin is the master-regulator molecule in tissue and serum iron metabolism. ^149^ This hormone acts on the transmembrane ferroportin molecule, regulating the iron inflow/outflow, hence regulating serum iron and TIBC, together with ferritin concentration.

On one hand the finding of a hyperconcentration of hepcidin likely reflects the inflammatory state in these patients; on the other hand the encountered molecular similarity between the tail of the viral spike protein and the hepcidin hormone may contribute to the overall ferritin rise. ^150^

In presence of a hepcidin-mimicking action by the spike protein on the iron metabolism, it is argued that an over-blocking action on ferroportin may occur. The consequent iron accumulation in the tissues, with blood iron deprivation, may contribute to the findings of hyperferritinemia, hypoferremia and low TIBC in most COVID-19 patients, especially at later/worse stages. ^8^^,^^151^

Together with the occurrence of cell membrane morpho-functional alterations caused by external stimuli, viroporin activity represents another general viral pathomechanism which may contribute to the intracellular cation overload. By means of viroporin channeling action towards the cell membrane, cations (calcium mainly) may tend to enter the cytoplasm, while on the other hand viral replication and subsequent viruses’ extracellular release is facilitated. ^152^^–^^154^

In COVID-19 these viral proteins were shown to respectively oligomerize and accumulate in the endoplasmic reticulum and in the Golgi apparatus; subsequently they can interact with the VGCC, based on the level of cell transmembrane charge potential, which ultimately leads to an alteration of the same cell membrane electro-chemical activity. ^155^^–^^158^

A few channels may be subsequently opened by viroporins from the inside of cell membrane, thus favoring the passage of extracellular cations into the cell and subsequent virus expulsion which results in NLRP3 inflammasome activation and cell apoptosis. ^155^^–^^157^

A number of viroporins have been described in coronaviruses and more specifically in SARS-CoV-2 infection, being protein E (from the envelope), ORF-3a and ORF-8a the most relevant ones. ^63^^,^^155^^–^^157^ The ion-channeling action of these viroporins exacerbate calcium/iron intracellular accumulation, tending to reduce the transmembrane voltage furthermore. ^156^^,^^159^ These complex phenomena significantly condition both SARS-CoV-2 virulence and the microenvironment of host cell, thus contributing to the final cell ferroptosis.

Cellular bioenergetics undergo major re-arrangements during viral infections; mitochondria represent the core-organelles for cell energy production and they typically undergo major changes in aging and in chronic degenerative diseases. ^160^^–^^162^

In general, the whole cell metabolism is strictly regulated by mitochondria activity and, equally, mitophagy is the driving process of cell apoptosis. ^163^ SARS-CoV-2 was shown to interfere significantly with mitochondrial activity, ^1^^,^^162^^,^^164^^–^^166^ which results in an increased blood level of a few specific biomarkers related to mitochondria oxidative stress, such as lactate, free radicals and LDH. ^1^^,^^164^^,^^166^^–^^170^ Past literature showed that the deregulated pathways of cation (iron and calcium mainly) metabolism invariably induce a relevant mitochondria degeneration, with altered mitophagy. ^35^^,^^171^^–^^176^

The complex degenerative sequences occurring in the infected cell, influenced by cation overwhelming concentration, may lead to several deranged biochemical pathways and ultimately to a specific type of apoptosis, named ferroptosis. The term ferroptosis refers to a peculiar form of cell apoptosis and it has been introduced in 2012. ^177^

This degenerative process takes place in case of excessive intracellular iron accumulation (under the form of ferritin and hemosiderin). Ferroptotic mechanisms are mostly based on lipoperoxidation and on mitochondria degeneration; they are considered of extreme relevance in a series of chronic degenerative diseases (such as neurodegeneration and cancer), ^178^^,^^179^ and more broadly in many biochemical pathways which are proper of cell senescence, ^180^ but also of viral infections. ^181^^,^^182^

An increasing number of articles have been showing the pivotal role of ferroptosis in the pathophysiology of COVID-19. In fact, based on the evidence above, the SARS-CoV-2-induced intracellular cation overconcentration may easily lead to a progressive deregulation of mitophagy, with a documented consequent ferroptosis-based cell degeneration in later stages and in worst scenarios. ^1^^,^^2^^,^^15^^,^^18^^,^^183^^,^^184^

Viruses were shown to generally alter iron metabolism, inducing somehow a form of iron deposition in the host cells; COVID-19 features a similar cell condition, which may be considered a sort of evolutionary and protective mechanism for viruses to survive and proliferate. ^185^^–^^190^

The high levels of intracellular calcium and iron in these patients, especially in later stages, significantly contribute to a number of dysfunctional metabolic pathways, both at mitochondrial level and at greater cell level. Cations notoriously undergo a strict homeodynamic interaction, influencing each other as to the intra/extra-cellular dynamics and concentration. Their accumulation in the affected cells is synergistically permitted by a series of mechanisms, which have been detailed in the scientific literature (see above) and will be highlighted furthermore in this paper.

In COVID-19 deceased patients different types of tissues are affected by iron deposition at autopsy examinations, regardless of virus localization. ^191^^–^^194^ More specifically, the post-mortem examinations documented iron-overloaded reticuloendothelial system, bone marrow, liver, lungs and generally iron-laded macrophages (hemophagocytosis). In fact, in association with the typical immune-mediated pathologic findings (e.g. leukocyte infiltrate etc.), hemophagocytosis represents another extremely common finding in bone marrow of critical or deceased COVID-19 patients, which may reinforce the hypothesis of a frequently occurring macrophage activation syndrome in this disease. ^195^^,^^196^

With reference to the pulmonary involvement in viral diseases, it was proven that iron and calcium altered homeostasis in lung cells may represent one of the factors to explain the onset of this interstitial infective-inflammatory phenomenon, which is characterized by alveolar macrophages laden with ferritin. ^1^^,^^197^^,^^198^

Obviously, intracellular cation accumulation in COVID-19 patients is also the result of the virus-based immune-inflammatory processes. Beside this explanation, we have highlighted that other SARS-CoV-2 pathomechanisms, based on cell membrane dysfunctionality and cations, may represent the core culprit of this disease.

In fact, virus interaction with cell membrane receptors, namely ACE 2, CD147 and SA molecules, proved to disrupt the whole cell membrane metabolic activity, through a profound deregulation of the transmembrane electric potential. The consequent intracellular cation accumulation and cell morphology/function alterations end up in the ferroptotic process, which likely represents the unifying terminal pathway at the root of the multi-organ attack operated by this virus.

## 3. Unifying pathophysiological hypotheses

Based on structural and functional studies, it has been highlighted that SARS-CoV-2 spike protein binds optimally three main host membrane receptors: ACE2, SA molecules and CD147 ^1^^,^^58^^,^^59^^,^^78^^,^^108^^,^^199^; similarly, it has been ascertained that the viral spike protein features an aminoacidic polybasic structure, which allows its functional processing by the human hydrolases and furin enzymes, so to favor the final anchoring of the virus to the cell membrane.

More specifically, the human cell transmembrane protease serin 2 (TMPRSS2), codified by the TMPRSS2 gene, contributes to the division of the spike protein in subunit S1 and subunit S2. ^200^

Similarly, the viral interactome with human cell membrane includes specific furin activity addressed to S1 subunit which exposes the RBD part. ^201^ In fact, furin cleavage is necessary to allow the exposure of the fusion sequences of the spike protein with cell membranes, which preludes to virus entry in the cell. The viral RBD is unique in terms of affinity to the molecules of SA which are found on cell membrane glycoproteins and on ACE2 and CD147.

The molecular and computational analyses of RBD/human receptors interaction have shown that single ACE2 target would not suffice to explain the great diffusion and entry capability of this virus. Thus, the viral interaction also with SA and CD147 represents a fundamental additional route to explain the multi-tissue diffusibility of this virus.

The documented deranged cation (iron/calcium) metabolism is a consequence of the viral interaction with the cell membrane receptors and with the ion-channels, as we have previously described and will detail further.

Iron homeostasis can be profoundly altered by different types of infections and by the concomitant inflammation. Similarly, hepcidin production is increased by inflammatory cytokines, especially IL-6 and IL-1b, which are typically over-released in more advanced stages of COVID-19, during the so-called “cytokine storm”. ^202^

Hepcidin essentially downregulates ferroportin and therefore determines hypoferremia and the sequestration of iron at cellular (macrophage *in primis*) level, leading to the typical anemia of any inflammatory state.

A growing body of evidence highlights how serum levels of iron, TIBC, ferritin, C-reactive protein and hepcidin tend to correlate with the severity of inflammation and, specifically with the prognosis of COVID-19. ^1^^,^^7^^–^^14^^,^^203^^–^^206^

As a reminder, it is acknowledged that the increase of blood ferritin expresses a compensatory mechanism to neutralize free circulating heme and Fe3+; in fact, the latter contributes also to the formation of methemoglobin, which would decrease hemoglobin O2-carrying function, hence deteriorating hypoxia furthermore in these patients.

A series of deranged metabolic pathways may take place at the root of the altered cation intracellular overload and of the multi-organ ferroptosis: a) altered ion (calcium and iron mainly) channel function, via cell membrane morpho-functional electro-chemical alterations and via viral viroporin channeling activity; b) higher hepcidin level, as result of the viral pro-inflammatory action and possibly via a hepcidin-mimicking action of the viral spike protein; c) viral invasion through the binding of SA molecules, ACE2 and CD147 of multiple types of cells, including erythrocytes/erythroid precursors, endothelial cells and macrophages, with relative hemoglobin and iron deregulated metabolism.

Here below we review in detail the main interplaying factors which intervene in COVID-19 cation/ferroptosis-based pathophysiology.

### a) Sialic acid

In SARS-CoV-2 interactome with host receptors, SA component of membrane glycoproteins and of receptors is considered of utmost importance due to its ubiquitous location. This molecule represents a fundamental mediator of cell metabolism derangement in several chronic degenerative diseases ^207^^,^^208^

The primary event in COVID-19 infection is the attachment of the virus particle to the surface of the host cell, which is mediated also by sialylated cell surface receptors on several types of cells, mainly in the respiratory tract in humans. Viruses interacting with humans preferably show a α2,6 binding to host cell receptors. ^99^

Recent experimental evidence has shown the ability of the SARS-CoV-2 Spike protein to bind SA molecules embedded in the membrane glycoproteins. In view of its negative charge, SA has a role in the determination of cell membrane electrical charge and in the consequent binding to the electrically positive charged spike protein S1. ^209^^,^^210^

Although the spike protein binding region has been identified in its N-terminal domain, this viral part is considerable as a dipole from an electrical point of view. In fact, due to the different aminoacidic composition, S1 segment (the so called RBD) has an overall positive charge, with 111 positive and 99 negative amino acids respectively; conversely S2 has a negative charge. ^209^

It is known that human cells have a negatively charged membrane, mostly due to SA molecules, to the ion pump activities and to the extra/intra-cellular pH. The average cell membrane resting potential is around -40/-70 mV. ^211^ Beyond this value expressed in mV, a more correct expression of this potential should be in (pico-)siemens (1/ohm), being this measure unit more reliably related to the conductance of the cell membrane. ^212^^,^^213^

Electrical properties of cells strictly relate to physiology and pathology, and literature data have clearly shown that microbes alter ion channel activity, cytoplasm activities and deformability. ^213^ Furthermore, scientific research has extensively documented the importance of SA in cell membrane interaction specifically with outer organisms or molecules. ^214^^,^^215^

From this point of view, erythrocytes were specifically investigated to detect their possible deformation when under pathogen attack or under specific biophysical/biochemical stressors; a different resistance and cell rigidity was found following to membrane and cytoplasm electrical changes. ^97^^,^^216^^,^^217^

Several publications have examined the virus-host cell interactome, elucidating the determinant role of SA for SARS-CoV-2 entry in the cell. ^6^^,^^80^^,^^83^^–^^85^ In fact, virus attack is mediated by the contact of the S1 subunit with the SA molecules; as a result of this docking, a dipole is formed, including SA negatively charged and S1 positively charged; this involves a variation of the local membrane potential. In view of the formation of numerous similar dipoles, the overall cell membrane potential undergoes remarkable electrochemical variations, lowering the total negative charge. This fundamental SA-based membrane change contributes to cation dysmetabolism, through a deregulation of ion channeling activity, as it was shown in numerous publications concerning electro-chemical physiology of human cell membrane ^218^^–^^220^

As to COVID-19 pathophysiology, it has been documented that the virus attack to cell membranes occurs starting through the formation of a fusion nucleus, derived from the spike protein and the host cell membrane; more specifically electrostatic bonds and hydrogen bridges favor this preliminary step.

The attacked human cells subsequently release the hydrolases (especially the TMPRSS2), which in turn cleave the spike protein creating the subunit S1 and S2, probably at the level of the aminoacidic interval 681-684, ^221^ or at the level of 861-865 segment. ^222^

On the other hand, human furins unveil the RBD in S1, which is then ready to dock with the cell membrane receptors. The subsequent transmembrane docking occurs between the positively charged [N(+) terminal] S1 RBD to the [C(-) terminal] receptors (ACE2, CD147, SA). ^209^^,^^223^^–^^226^

As the virus RBD interaction with the sialylated cell membrane glycoproteins/receptors (ACE2 and CD147) results in a lower electro-negative membrane potential, the consequent dysfunction of the membrane ionic pumps conditions the whole pathophysiology of COVID-19.

The past investigation of cell physiology clearly showed that SA concentration and its disposition in cluster (hence the resulting membrane potential) strictly influence VGCC function. ^218^^,^^219^ Once the electro-chemical variations mentioned above occur, the greater opening of the VGCC lead to iron/calcium hyperconcentration inside the cell. ^220^

With relevance to iron metabolism, it has been documented that any perturbation of the membrane potential and of ion channels lead also to an altered ferroportin function. ^227^ In COVID-19 this possible functional alteration of the membrane, and specifically of ferroportin, contributes furthermore to the intracellular ferritin accumulation, also in view of the deregulated axis with hepcidin.

Beside the cell membrane functional electro-chemical changes caused by the interaction between S1 and S2 subunits with host cell, also morphologic changes of membrane and cytoplasm may occur. For example, in septic conditions membrane SA concentration tends to decrease, which leads RBC to become spherical and to decrease their intercellular repulsion; these variations tend to increase blood viscosity and this finding constitutes one of the pro-coagulant elements which can be encountered in these patients. ^228^

Analogue morphologic changes of the whole cell may occur in many more tissues: this phenomenon depends upon the *milieu* pH, the membrane potential charge, the concentration and conformation of the receptors, which also influence the osmotic gradient. ^100^

### b) CD147

Together with SA, also CD147 transmembrane receptor glycoprotein plays a role in COVID-19 pathogenicity. ^1^^,^^55^^,^^56^^,^^64^^,^^229^^–^^232^ The receptor activity of this sialylated transmembrane glycoprotein has been studied in a large number of diseases. ^68^^,^^69^^,^^233^^,^^234^

In case of SARS-CoV-2 attack, numerous effects have been documented in tissues and organs where ACE2 receptors are few if present at all (e.g. erythrocytes, leukocytes, platelets, endothelium, cardiomyocytes, neural cells, kidney). ^63^

Of interest, CD147 is the receptor malaria parasite uses to enter cells. As COVID-19 infection rate seems to be much lower in those countries where malaria is more endemic, ^235^^–^^237^ it is argued that some competition for the same receptor may likely contribute to explain the low infection rate in these African populations. Similarly, in these countries sickle cell anemia is widely diffused, which is considered a protective evolutionary mechanism. Also, this phenotype may be a possible additional protective factor from COVID-19 in the same populations of malaria areas. ^237^^–^^239^

A long series of publications have regarded the possible SARS-CoV-2 interaction with erythrocytes and the consequent hemoglobin dysfunction, with release of free circulating hemoglobin/heme. ^21^^,^^22^^,^^128^^,^^132^^,^^240^^,^^241^ More importantly, a few researchers highlighted clear erythrocyte membrane alterations in COVID-19 patients. ^4^ These and previous data seem to demonstrate furthermore the CD147/SA-based interaction of SARS-CoV-2 with RBC which, at least partially, explains the consequent hemoglobin/iron cycle alteration. A preliminary report has also evidenced a significantly higher CD147 surface expression (and an increased oxidative stress) in erythrocytes of COVID-19 patients in comparison to normal subjects. ^242^

Actually, higher RDW and lower hemoglobin figures have been highlighted, especially at later stages; moreover RDW is considered an important prognostic factor in this disease. ^243^^–^^246^ High values of RDW typically indicate an altered synthesis of RBC precursors, and a number of authors confirmed this deranged erythropoiesis also in COVID-19. ^1^^,^^74^^,^^247^^,^^248^

Further to the documented role of CD147 in the viral attack to the cell membrane, this receptor was found to interact with cyclophilin A, which is one of the human proteins used by SARS-CoV-2 for its intracellular replication. This interaction regulates cytokine secretion and chemotaxis of inflammatory cells, which facilitate the infection of the host cell. ^64^^,^^249^^,^^250^

### c) Viroporins

As previously elucidated, viral encoded membrane pore forming protein (viroporins) may play a relevant role in the intracellular viral attack. Intra/extracellular pH and the transmembrane potential charge (both of them influenced by SA molecule activity and by the ion concentration) impact the cell membrane depolarization of these ion-channeling viroporins.

Viroporins are chronologically activated after the virus entry and do facilitate viral replication; they are known to exert a channeling action from the inner side of the host cellular membrane directed towards the extracellular space. ^152^^,^^153^ The modulation by viroporins on the opening/closure of multiple pores in the cell membrane tends to regulate cation transmembrane movement, but also endoplasmic reticulum cation release. ^5^^,^^251^

In fact, these small hydrophobic proteins are encoded by the virus and are oligomerized in the membrane of host cells, leading to the formation of hydrophilic pores. This activity disrupts several cellular functions, including membrane permeability, calcium homeostasis, membrane remodeling and glycoprotein trafficking. ^152^^,^^153^

One of the main functions of viroporins during viral replication is to participate in virion morphogenesis and release from host cells. Furthermore, some viroporins are involved in virus entry and genome replication.

The existence of viroporins was originally suggested following the observation that virus-infected cells become permeable to ions and small molecules. New members of this expanding family of viral proteins have been described, from both RNA and DNA viruses. These proteins are crucial for viral pathogenicity due to their involvement in different stages of the viral life cycle. Their main function is to participate in the assembly of viral particles and their release from the infected cells. Typically, deletion of a viroporin-encoding gene from a viral genome dramatically reduces viral progeny formation and viral pathogenicity, emphasizing the essential role of these proteins in the viral-human cell interaction. ^152^

SARS-CoV-2 viroporins, mainly type E, act to re-potentiate the channeling action and ultimately the entry of cations and new viruses in the cell. In fact, after the early depolarization of cell membrane due to S1 (especially) and S2 docking, virus entry and calcium/iron influx take place through ion channels (e.g., VGCC). Once the cation-enriched gradient grows up, the trans-channel ion movement from the extracellular space tends to decrease. Intracellular viroporin channeling action tend to immediately re-activate virus and cation entry against the gradient, thus ultimately favoring virus intra/extra-cellular diffusion and ultimately cell ferroptosis.

### d) Calcium and iron dysmetabolism

A number of cations show a low blood level during COVID-19 course. ^32^ Interestingly, calcium represents one of the most investigated minerals in this disease and hypocalcemia has been repeatedly reported in these patients, ^26^^,^^252^^–^^254^ especially at later stages. ^24^^–^^28^^,^^30^^,^^33^

The low serum calcium level lays for a probable progressive intracellular deposition of this mineral, as no specific hypercalciuria has been detected. The prognostic role of hypocalcemia, ^27^^,^^29^^–^^31^ together with the significantly dysregulated calcium metabolism/signaling in COVID-19 patients, have been investigated in two recent reviews. ^255^^,^^256^

Physiologically, calcium intra/extra-cellular balance and homeodynamics are strictly depending on the so called VGCC. The latter represent highly regulated cell proton pump mechanisms. Excessive intracellular calcium has been related to several chronic degenerative diseases, mostly linked to peroxynitrite accumulation and oxidative stress. ^175^^,^^257^^,^^258^

In COVID-19, the documented hypocalcemia and the consequent intracellular hyperconcentration of calcium may originate a series of derangements, mostly through mitochondria deregulation, as already highlighted in other diseases. ^259^^–^^261^

Of interest, it was shown that a specific genetic susceptibility linked to VGCC may predispose to Kawasaki autoimmune disease, ^45^ which may occur among children in the current pandemic. ^262^ Neuronal cells may similarly suffer from a deregulated calcium and iron concentration, as these cations intervene in the conductivity process; overall, neuro-sensorial disturbs, including loss of smell and taste, have been extensively reported in the vast majority of COVID-19 cases. ^263^^–^^266^

Calcium is essential to most viruses as to their processes of entry, replication and diffusion. ^34^ For example in case of SARS-CoV-1 infection, a calcium-dependent transmembrane invasion was demonstrated ^267^ and it was proven that generally any alteration in host cells calcium homeostasis reflects upon viral pathogenicity and diffusion. ^35^^,^^268^

More in detail, it has been ascertained that viral replication is also based on a sort of hijacking of a few cation-based host cell processes, which mainly pertain mitochondria. This mitochondria deregulation results in a number of biochemical degenerative processes, such as NLRP3 inflammasome activation and a deregulated cell apoptosis (ferroptosis namely). ^34^^,^^269^

As previously reported, a similar mitochondria “kidnapping” and degeneration due to SARS-CoV-2 infection has been described and hyper-concentrated iron and calcium on one side mediate this detrimental effect, ^2^^,^^166^^,^^270^ on the other side stimulate ferroptosis. ^35^^,^^44^^,^^271^

In normal conditions body iron is mostly located in the prosthetic group of heme; hyperferritinemia reflects excessive iron availability, but also it could hypothetically derive from damaged tissues. ^7^^,^^272^ Thus, in order to assess iron metabolism properly, additional biomarkers (such as TIBC and other transferrin-related markers) should be investigated, so to have more surrogate findings which mirror iron deposition and free circulating iron. ^273^

In these conditions of hyperconcentration of this metal inside the cell, free cellular iron (Fe3+) can easily form free radicals, for example through Fenton and Haber-Weiss reactions, thus a series of detrimental biochemical pathways may be activated, including pro-coagulative cascades. ^274^ The same transferrin molecule has been recognized as an important pro-coagulant factor in COVID-19, ^275^ probably due to its interaction with several clotting factors. ^276^

Of interest, the “protective” mechanism of iron sequestration under the form of ferritin (especially in macrophages of lungs, liver etc.) can impair erythropoiesis and new hemoglobin production, which may complicate anemic hypoxia in patients with COVID-19. ^273^

Physiologically, in mitochondria iron represents a fundamental co-factor of several enzyme-based reactions; furthermore Fe2+ is transformed in its bioavailable form by a cluster iron-sulfur (Fe/S) along the heme synthesis pathways. ^277^ When iron is in excess, its altered pathways lead to a much higher production of free radicals; furthermore heme (thus hemoglobin) formation decreases, originating a sort of sideroblastic anemia in the case of COVID-19 patients. ^1^

Ferritin overloading cells leads to ferroptosis, which could be considered also an evolutive protection mechanism, aimed at reducing both free serum iron and its availability to viruses and other pathogens. Likely, ferroptotic mechanisms are upregulated in similar conditions of ferritinophagy, high amounts of free cell iron, deregulated hepcidin/ferroportin axis and especially exaggerated cation entry.

### e) Cell membrane electrochemical changes and ion pumps

Following to the described morpho-dynamic viral interaction with cell membrane, and after the significant reduction of the transmembrane electronegative potential, the charge change relevantly impacts ionic pump regulation. The docking between S1 and S2 with membrane receptors (S1) and phospholipid layers (S2), as specified above, are mostly based on the binding with SA molecules and it leads to an altered ion channeling activity. VGCC modification usually takes place, permitting the excessive entry of calcium and, consequently, of Fe2+ ions. ^63^^,^^218^^–^^220^ Lastly, as previously documented, the subsequent intervention of the viroporins contributes to create new pores in the cell membrane, allowing further cations to enter, additionally facilitating virus diffusion.

Biophysical studies have recently highlighted that SARS-CoV-2 itself owns its electrical properties and generates an electro-magnetic field (EMF), which can interact with the polarity of the host cell membrane; this possible interplay urges a better understanding of the whole electrical and biochemical process occurring during the infection. ^278^

The detrimental chain of biochemical events caused by cation intracellular overload, such as Fenton reaction with Fe3+ formation, H2O transformation into hydroxyl free radical and NO transformation into the toxic ONOO (peroxynitrite) compound has been described in COVID-19 as well, especially concomitant to cytokine storm. ^279^ Moreover a few authors have advocated an involvement of hemoglobin denaturation, with free heme/iron release, at the root of the free radical production. ^3^

Lastly, the intracellular migration of iron and calcium ions is responsible for the activation of the inflammasome NLRP3, ^280^ which typically contributes to worst scenarios of this disease.

Altered ion exchange ultimately generates lipoperoxydation and oxidative stress (the root of ferroptosis), consuming glutathione and deeply damaging mitochondria. The consequent dysregulated mitophagy accelerates apoptosis of the infected cells. ^178^^,^^179^

A computational study has elucidated that host cell membrane interaction with the SARS-CoV-2 viroporin channeling action features a cation selectivity, being sodium, potassium and calcium the most facilitated ions in the transmembrane movement; moreover, the authors showed that transmembrane voltage influences the pore dimension and the transition rate (thus the intracellular accumulation) of cations. ^158^ Similar findings had been discovered for viroporins in general, thus confirming the importance of the electrochemical gradient of cell membrane in ion balance. ^152^^,^^281^^,^^282^

Also virus fusion, invasion and replication are greatly enhanced by the progressively more favorable intracellular cation-enriched microenvironment. ^158^

As mentioned above, the blood levels of other cations, such as potassium and sodium, have been shown to decrease in COVID-19 patients. Generally coronaviruses, and more specifically SARS-CoV-2, have been found to exert a channeling activity via viroporin E and ORF3a, ^63^ which leads to an intracellular K+ overload. ^155^^,^^283^ Of note, as calcium inflow is physiologically linked to potassium movement, a sort of vicious circle may arise, inducing an augmentation of the calcium concentration inside the cell.

As we have seen, iron and calcium influence mitochondrial function and several metabolic processes. Additionally, cellular iron stimulates calcium signaling and vice-versa, which impacts also ferroptosis. ^44^^,^^271^^,^^284^

Through VGCC, also iron (and other metals) may enter cells, as biophysical studies have documented. ^285^^–^^291^ Neurodegenerative diseases are paradigmatic examples of cell intoxication with iron and other metals, through VGCC dysfunction, ^284^ and calcium channels were shown to favor glutamate accumulation in neuronal diseases, giving rise to the so-termed “oxytosis”, which originally described ferroptosis. ^292^

The virus-based intracellular cation engulfment affects also the two-pore cation channels in the lysosomal membrane, thus reducing the endo-lysosomal “digestive” function against microbes. ^293^

In the light of the evidence above, scientific research is reserving a major attention to the relationship between iron and calcium on one side, and the virus-host cell membrane electro-chemical interactions on the other side. ^106^^,^^156^^,^^273^^,^^278^^,^^294^

### f) Hepcidin, ferroportin and transferrin

Hepcidin is the master-regulator peptide hormone in iron metabolism. Basically, hepcidin is to iron what insulin is to glucose and an alteration of its interaction with ferroportin has been linked to a series of chronic degenerative diseases. ^149^ From the first reports on hepcidin discover, ^295^^,^^296^ the understanding of iron homeostasis has become more and more precise, revealing a large number of metabolic activity. Iron circulates inside-outside the cell in different manners: introduction by extracellular transferrin capitation and internalization, or by divalent metal channel DMT1. ^297^

Iron metabolism, both regarding intestine absorption and RBC catabolism, involves transferrin and ferroportin for blood transport and extracellular release respectively. Hepcidin strictly regulates ferroportin activity. When the transferrin saturation capacity is exceeded, ferric iron ions can be released in the bloodstream, with noxious repercussions systemically. In COVID-19, higher values of interleukins and other factors specified above increase hepcidin activity, so to store the potentially harmful iron under the form of blood/cell ferritin.

Plasma levels of transferrin are dependent on iron requirement and availability. As in these patients hypoferremia generally occurs, higher values of transferrin have been concomitantly recorded, suggesting an upregulation response to the infection, with possible pro-coagulative repercussions as well. ^275^

It is known that the two linked phenomena of high hepcidin activity and low ferroportin efflux, which take place in these infected patients, decrease the amount of iron bound to transferrin, whereas decreased hepcidin and high ferroportin activity may be associated with an increased transferrin saturation. ^273^

Intracellular iron exists in two forms, Fe2+ and Fe3+; as Fe3+ is the toxic form more available to generate free radicals, normally cell embeds this ion in the ferritin molecule inside specific vesicles. A minority of Fe3+ is deposited under the form of hemosiderin. This equilibrium between the two iron forms is unbalanced in presence of excessive hepcidin activity. Also, hyper-formation and storage of ferritin, based on the hyperconcentration of hepcidin molecules, requires a relevant consumption of ATP, with consequently increases mitochondrial dysmetabolism.

Once in the plasmatic and cytoplasmic compartment, iron is a labile ion that could interact with numerous molecules, stimulating, and inhibiting different processes. A homeostatic mechanism exists with regards to intra/extra-cellular iron concentration. Hepcidin has the role of blocking and internalize the transmembrane ferroportin, preventing iron efflux. If this excessive intracellular uptake is combined with a reduction of efflux, an overcharge of intracellular labile iron with ferritin hyper-concentration and formation of toxic free radicals may occur. ^298^

With concern to the immune-inflammatory derangement which takes place in COVID-19, a direct link has been highlighted between viral ACE2 receptor internalization and hepcidin-IL6 activation through the NF-kB system. ^205^^,^^280^

This mechanism suggests the occurrence of a vicious cycle, where transferrin uptake and hepcidin overexpression cause a persistent activation of NF-kB as a consequence of the intracellular iron augmentation. Literature data put in evidence that also CD147 receptors stimulate NF-kB system, ^299^^,^^300^ suggesting that both ACE2 and CD147 act on the same system and consequently generate the same cascade of events within the inflammasome NLRP3. More in general, inflammasomes include a class of intracellular proteins involved in inflammatory reaction in most chronic and acute inflammation processes, such as obesity, diabetes, stroke, cancer, ^301^ hypoxia and thrombosis. ^302^

Similarly, it was proven ^303^^,^^304^ that the NLRP3 inflammasome is directly activated and related to the NF-kB system and, more interestingly, to the intracellular labile iron, suggesting a complex interaction with ACE2 and CD147 receptors in COVID-19 infection.

Beyond the strict relationship between (chronic) inflammation and hepcidin/ferroportin axis activity, with an anemia pattern and an increased level of circulating hepcidin, ^305^^,^^306^ COVID-19 has been associated with a marked increase of serum hepcidin level in worse patients. ^203^^–^^205^

As previously described, early publications about COVID-19 ^1^^,^^150^ reported about a similarity between the aminoacidic sequence of SARS-CoV-2 spike cytoplasmic tail and the hepcidin molecule. Hence the virus could perhaps block ferroportin-based iron extracellular transport and an accumulation of iron inside the cell could occur.

Note that hypoferremia and hyperferritinemia along the course of the disease should deactivate hepcidin activity and reduce its blood levels. As this is not the case in the studies cited above, the viral pathomimicry could at least partially justify the iron deregulated axis in these patients.

### g) Hemoglobin, heme

Nearly 75% of human iron is contained in hemoglobin; in view of the iron deranged metabolism in COVID-19, a number of articles have investigated a possible hemoglobin denaturation in these patients ^1^^,^^3^^,^^22^^,^^63^^,^^126^^,^^128^^–^^130^ Though no sound evidence of a direct hemoglobin viral attack has been documented so far, free circulating heme has been described in a few papers ^132^^,^^240^^,^^241^; moreover, some recruitment of hemoglobin and its metabolites (hemin and protoporphyrin IX) by a few SARS-CoV-2 proteins was documented in a computational biology study. ^241^

An analogue scientific debate is underway about the alteration of the dissociation curves of hemoglobin in these patients, regardless of whether hypoxia may not be regarded as solely generated by pneumolysis/lung disease. ^145^^–^^147^^,^^307^

### h) Potential environmental, genetic and microbiome susceptibility factors

Beside AB0 group and sickle cell conformation, several other factors may predispose to SARS-CoV-2 pathogenicity.

Specific chromosome variants or sequences have been linked to worse prognosis, but also genetically-determined hemochromatosis is being considered of some importance. In fact, liver biopsies in seriously ill patients, or during autopsies, have shown a remarkable intracellular iron accumulation, which clearly reminds hemochromatosis histopathologic features. ^193^ Similarly, a ferroptotic pattern has been encountered in a fatal case of myocarditis ^192^ and a typical pattern hemophagocytosis with iron-laden bone marrow cells was documented in a large series of autopsies. ^196^

Hereditary hemochromatosis is characterized by an accumulation of ferritin in several tissues, which may be clinically asymptomatic or less pronounced in heterozygosis type, more aggressive in homozygosis.

The incidence of heterozygote hemochromatosis has been reported as high as 5.4-13.5% in the general U.S.A. population ^308^ and, more specifically, a review ^309^ found the following prevalence percentages in the general population: 5% as compound heterozygotes (C282Y/H63D mutation), 1.5% homozygous for the H63D mutation, 3.6% were C282Y heterozygotes, and 5.2% were H63D heterozygotes. With reference to world areas, the frequency of the C282Y heterozygosity ranges from 9.2% in Europeans to much lower figures in Asia, Africa and Middle East. Conversely, the H63D carrier frequency was documented as 22% in European populations.

In case of SARS-CoV-2 contagion, the subject with hemochromatosis may undergo a more complex course; being this disease latent in the vast majority of the individuals, it could be of interest to investigate its presence in the patients showing an atypical (e.g. young, or apparently healthy subjects) complicated infection. ^1^^,^^310^^,^^311^

Environmental factors have equally been called into question as possible disruptors in the course of this infection. In the past years a number of articles have evidenced in animals and humans the possible role of EMF on the dysfunctional pathways of VGCC, on peroxynitrite formation, oxidative stress and on other biochemical pathways at the root of cation dysmetabolism. ^312^^–^^322^

Additionally, in the last decades scientific literature has documented the influence of EMF on viral activity, erythrocyte/viroporins metabolism, mitochondria and immunity. ^323^^–^^327^

The possible interaction of 5G technology with human health is being debated as well, raising contrasting speculations and evidence, mostly due to the limited knowledge about this relatively new EMF typology. ^328^^,^^329^ More recently, a few specific papers focused on the possible role of peculiar types of EMF in SARS-CoV-2 pathogenicity, ^330^^,^^331^ and a recent publication has presented some data about the detrimental interaction of radiofrequencies specifically with VGCC and with cation metabolism, erythrocytes, hemoglobin, coagulation, immune system and oxidative stress. ^332^

In view of the role of both the cell membrane electro-chemical changes and the cations in COVID-19, some more research could be performed to assess any possible inter-relationship of polluting environmental factors with VGCC, cellular membrane and cytoplasm ionic changes.

Microbiome is another potential element of interest in this infection. Microbiome regulates a large number of bodily biochemical pathways and it is considered of utmost importance in several chronic degenerative diseases and, more generally, in cell senescence. ^333^^,^^334^

A few authors have highlighted an analogue specific role of microbiome in COVID-19, some of them focusing on the pathogenicity of lipopolysaccharides (LPSs); LPSs are known byproducts of gram-negative bacteria which derive from an altered microbiome and gut permeability, inducing endotoxemia. ^335^^,^^336^ A strict relationship between LPSs and ferroptosis has been already established in past literature ^337^^–^^340^; similarly, it was documented that LPS interaction with spike proteins may be mediated by SA receptor activity, ^341^ which seems to reinforce the role of SA molecule in the LPS-mediated ferroptotic processes in these specific patients.

In fact, a high binding affinity between LPSs and SA has been repeatedly demonstrated, ^341^^–^^344^ hence the complex interaction among LPS, SA and SARS-CoV-2 spike protein, with the resulting pro-ferroptosis pathways, might deserve more attention in the future scientific research.

### Summarizing the evidence and the hypotheses

Overall, the hyperinflammatory reaction which characterizes the worst scenarios of COVID-19 may be regarded as an expression of the immune-inflammatory derangement, which in turn could depend upon the cascade of the biochemical pathways that characterize intracellular cation accumulation and cell ferroptotic mechanisms. The very final detrimental degenerative cell events are then represented by excessive free radical formation (peroxynitrite above all), ^279^ glutathione depletion, membrane lipoperoxidation, mitochondria degeneration, partial metabolic shift to anaerobic glycolysis, marked activation of the inflammasome NRLP3 and NF-kB with subsequent inflammatory cytokine cascade.

At the very beginning of this sequence of pathomechanisms, it is possible to recognize the relevant role of the electrochemical changes induced by the spike proteins on the cell membrane, via glycosylated receptors and ion channeling alterations.

## 4. Discussion

The finding, now confirmed by many publications, of low levels of hemoglobin, serum iron and calcium, together with the very high levels of ferritinemia in critical or deceased patients, highlights the major derangement of cation metabolism in these patients. Similarly, higher figures of LDH, lactate and RDW express both the involvement of mitochondria, and some form of degeneration of RBC and of their precursors.

The intervention of the negatively charged SA, both on the receptors ACE2 and CD147 and on the sialylated membrane glycoproteins, proves extremely relevant in the mediation of the docking process with the spike proteins.

This docking interaction induces a decrease of the negative potential of the cell membrane and, as consequence, trans-membrane cation channels may alter their permeability so that, on a multi-tissue level, different types of cells undergo morpho-functional changes. Of course, the latter depend upon several other factors, including pH and osmolarity of the intra-extracellular space. ^100^

Hepcidin, as a result of both the hyperinflammatory state and of a possible mimicking action by the viral spike protein, remarkably contributes to ferritin and hemosiderin accumulation in the tissues, by reducing ferroportin activity.

Lastly, viral viroporins and other viral pathomechanisms contribute to the accumulation of the cations inside the cell, thus contributing to viral replication/extra-cellular release and cell degeneration.

The resulting calcium and iron hyper-influx impairs homeodynamics of a series of cellular (mitochondrial primarily) pathways, and also favors a dramatic free radical increase. Excessive ferroptosis represents the ending outcome of these degenerative changes in many tissues (e.g., lung, endothelium, heart).

Erythrocyte degenerative electrochemical changes of the membrane, with morpho-functional alterations (such as spherocytosis, altered erythropoiesis, hemoglobin dysfunctionality) may deserve some specific attention. In fact, iron metabolism strictly depends upon RBC and hemoglobin and several authors have documented some degree of (autoimmune-like) hemolysis, with an increased RBC destruction which takes place in the reticuloendothelial system and in specific organs (spleen, liver etc.). Lastly, some literature data point out the free heme circulation in patients affected by COVID-19.

**Figure 1.**
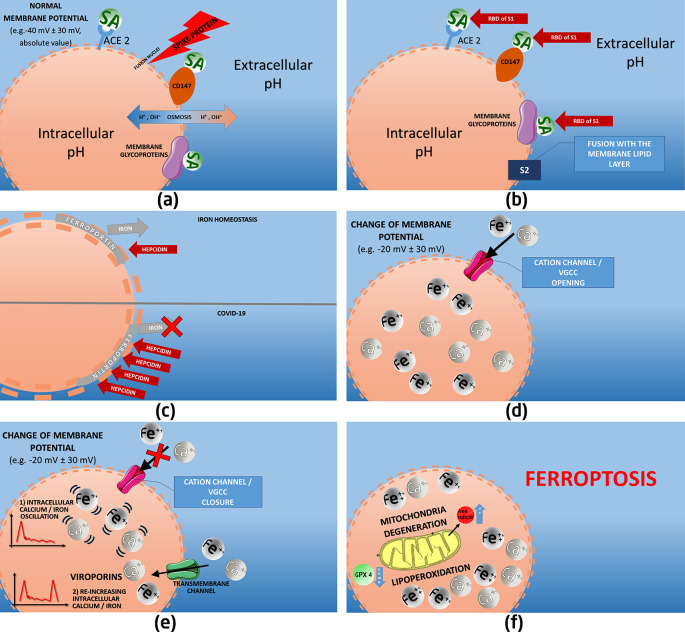
a) Docking approach of the spike protein with the host cell membrane, which presents three main receptors: ACE2, CD147 and Sialic Acid (SA); SA is expressed on the membrane glycoproteins and on the outer site of the other two receptors. b) The two subunits of the spike protein (S1 and S2), derived from priming and cleavage by hydrolases (TMPRSS2) and furins, contact host cell membrane: S1 receptor binding domain (RBD) attach ACE2 and CD147 through SA and directly SA on the membrane glycoproteins; S2 enters the lipid layer of the cell membrane. c) Upper part: normal hepcidin-ferroportin axis; lower part: COVID-19 situation, with hyper-concentrated plasma hepcidin molecules that bind the extracellular portion of the transmembrane ferroportin, thus blocking iron extracellular transport and favoring intracellular ferritin accumulation. d) Decrease of the membrane potential (less negatively charged), which is altered by the formation of several dipoles between SA negative and S1 positive; consequent opening of the cation channels, especially of the voltage gated calcium channels (VGCC), with intracellular entry of cations, primarily calcium and iron. e) Cell homeostasis, after cation entry, rapidly brings closure of VGCC and cation channels (1 intracellular oscillation with one peak of cations); simultaneous viroporin action of membrane channeling which brings opening of new channels and re-entry of cations (2 intracellular oscillation with a second peak of cations). f) Ferroptosis: excessive cation concentration, increase of free radicals, depletion of glutathione peroxidase 4 (GPX4), lipoperoxidation of membranes and organelles, mitochondria degeneration.

To summarize the probable events at the root of cation imbalance and ultimately of ferroptosis, we have reported the main pathological steps in Table 1 and in Figure 1.

**Table 1.** Main pathological steps of the SARS-CoV-2 attack to cell membrane and of consequent cell degeneration.

a) Initial attack of the spike protein at the host cell membrane; the latter is physiologically charged negatively and with a variable potential (around 40mV +/- 30 mV); the level of the negative charge depends on the number and on the type of the receptors. The main involved receptors are: SA on the membrane glycoproteins, ACE2 and CD147; SA molecules are expressed at the outer surface of ACE2 and CD147, which favor viral attack; b) Morphologic changes of the whole cell may occur: this phenomenon depends upon the *milieu* pH, the membrane potential charge, the concentration and configuration of the receptors, which also influence the osmotic gradient; c) Formation of fusion nuclei between the spike proteins and the host cell membrane by means of electrostatic bonds and hydrogen bridges; d) Release from the host cell of hydrolases (TMPRSS2) which cleave the spike protein separating the S1 subunit from S2, probably at the level of the aminoacidic interval 681-684; on the other hand, furins simultaneously unveil the RBD in S1; e) Fusion of S2 to the lipid layer of the plasma side of the cell membrane; f) Transmembrane attack of the positively charged [N(+) terminal] S1 RBD to the [C(-) terminal] receptors (ACE2, CD147, sialylated glycoproteins), with numerous dipole (S1 positive-SA negative) formation; g) Additionally, hyper-concentrated plasma hepcidin molecules bind the extracellular portion of the transmembrane ferroportin, thus blocking iron extracellular transport; h) As consequence of these synergistic events a change of the membrane electrical potential occurs, which leads to an opening of the cation channels, especially of the VGCC; cations, primarily calcium and iron, enter the cell and concentrate in the cytoplasm and in the organelles; i) Subsequent rapid closure of the VGCC occurs, due to cell homeostasis and re-balancing of the protonic gradient, while a simultaneous intracellular viral replication leads to the production of viroporins; these viral hydrophobic proteins exert a membrane channeling action, thus facilitating new entry of cations from the extracellular space against the gradient, also favoring replication and external release of viruses; j) Re-increase of calcium/iron influx and their intracellular accumulation, which leads to an extremely high oxidative stress, mitochondria degeneration, membrane lipoperoxidation, glutathione peroxidase 4 (GPX4) depletion, all of this configuring the terminal ferroptosis.

The pathophysiology sequence of the virus-host cell interaction which has been proposed above, is based on the basic knowledge of the spike protein attack to cell membrane ^209^^,^^223^^–^^226^ and focusing on SA, CD147, ACE2, hepcidin, viroporins and on the electrochemical changes happening on the membrane, with subsequent cation imbalance and ferroptosis.

Cell ferroptosis is the final step of the cascade described above. This specific cell death process is caused by a pronounced lipoperoxidative transformation of several cell components, with glutathione peroxidase 4 (GPX4) depletion; finally a deep dysregulation of mitophagy with mitochondrial degeneration occur, inducing an accelerated and deregulated apoptosis. ^345^

Of interest, other relevant biochemical pathways inherent to ferroptosis have been described, such as the ferroptosis suppressor protein 1 (FSP1) mechanism, ^179^^,^^346^ which is based on a ferroptosis-resistant proteic factor that acts protecting from GPX4 depletion. In fact, it was shown that FSP1 may increase the quote of ubiquinol using NAD(P)H as co-factor (starting from the oxidized form ubiquinone); hence, this cytoplasmic membrane pathway ultimately collaborates with GPX4 and glutathione to reduce the impact of ferroptotic pathomechanisms.

Most recently, another anti-ferroptosis defensive mechanism was discovered in oncology. Inducing ferroptosis in cancer cells is promoted as one of the innovative possibilities in oncological therapeutics; recently it was discovered that supplementation with dihydroorotate/orotate (the first is the substrate and the second is the product of dihydroorotate dehydrogenase (DHODH)) respectively mitigate or exacerbate ferroptosis and mitochondria lipid peroxidation. ^347^

How FSP1pathway relates specifically to COVID-19 pathophysiology has not been investigated yet, conversely a very recent review reported the outcomes of two preliminary in-vitro studies where DHODH inhibitors halted SARS-CoV-2 replication. The results of these studies led the authors to elaborate on the possibilities of a few re-purposed anti-ferroptosis drugs for the therapy of this infection. ^348^

Cell membrane altered polarization and the overload of cations can also affect blood coagulation, endothelial and neural functionality; more generally the electro-chemical events occurring at the cell wall significantly impact the metabolic pathways of a number of organs.

Most of the biochemistry alterations reported above are also linked to the relevant immune-inflammatory process which takes place in these patients. In fact, SARS-CoV-2 infection leads to the typical inflammasome activation, with NF-kB activation and interleukins/TNF-alfa increase; these pathways involve extremely higher hepcidin production with intracellular iron sequestration, which favors viral replication. However, in comparison to other inflammatory/infective processes, ferritin peaks higher figures and in a shorter time in the course of this disease. ^1^^,^^205^^,^^349^^,^^350^

Hence, ferritin is regarded both as a typical consequence and marker of the SARS-CoV-2-induced inflammatory cascade, and as a potential pathogenic mediator of the viral infection.

Furthermore, ferritin was found to have a different biochemical composition (higher protein component) when derived from inflammatory diseases, whereas in these cases it is expected a higher ferric component and a different composition in its two H and L subunits. ^351^ In fact, for a long time now, scientific research has shown how viruses tend to take control of iron metabolism and of VGCC, so to establish a preferential microenvironment for their growth and multiplication. ^185^^–^^188^

With reference to the possible therapeutic proposals targeting the basic pathophysiology steps elucidated above, literature highlights a number of options to address this cation dysregulation, based on chelation for example. ^15^^,^^187^^,^^352^ Equally, a large series of drugs and natural compounds have been tested, or advocated, to target the sialylated receptors, hepcidin/ferroportin axis and the cation channels deregulation.

More robust data are needed before drawing definitive conclusions about the role of intracellular cation accumulation in the onset and perpetuation of the inflammatory and immune-mediated processes of this infection. However, through this narrative review we aimed at addressing the electro-chemical pathomechanisms which are at the root of the viral attack, and we highlighted as well the host cell membrane morpho-functional changes which relate to cation imbalance.

Furthermore, newer insights were provided about the role of SA, the CD147 receptor, about the hepcidin-ferroportin axis deregulation and the erythrocyte/hemoglobin altered metabolism.

Some contrasting literature data were reported on the specific subjects’ different susceptibility to the viral attack, based on determined genetic mutations, blood groups/diseases, specific receptors expression. ^73^^,^^193^^,^^311^^,^^353^^–^^360^ Beyond a specific possible TMPRSS2-related predisposition on a genetic or epigenetic (e.g. diabetic and obese patients) basis, ^361^ the majority of these speculations refer both to the variability of the membrane expression of certain receptors (namely SA and CD147), and to the consequent cation dysmetabolism.

For example, past research has linked blood group to susceptibility to viral infections for a number of reasons, mostly connected to membrane receptors. ^62^

Basically, AB0 blood group glycans were found to modulate SA recognition on erythrocytes and it was reported that there are approximately 2 million AB0 glycan antigen sites on each RBC and 50 million SA molecules per erythrocyte. ^96^

Similarly to CD147 expression on RBC wall, also different SA molecules represent the terminal part of glycoproteins which are expressed in variable formulas and clusters on the surface of several kinds of cell, thus on the RBC membranes as well. Interestingly, SA has possible substituent groups, especially in position 5 of the carbon atom, which are of extremely variable nature. ^362^ This peculiarity remarkably modifies both the charge (which remains always negative) and the stereochemistry of the SA molecule from the primary to the quaternary conformational structure; additionally, further substitutions may occur in the overall glycoproteic molecule and, of importance, different concentrations of SA molecules have been highlighted in the various kinds of cells and individuals. ^107^

In a speculative review the possible protective and detrimental role of 0 and A blood group respectively, was linked to a lower (0 group) or higher (non-0 group) attack from innate immunity. This reaction would take place based on the different distribution of the erythrocyte membrane glycoproteins. ^111^

AB0 antigens are known to modulate cellular interactions with outer molecules, microbes and parasites (such as *plasmodium falciparum)*, not as ligands, but by stabilizing other glycans such as SA on the cell surface in clusters, thus influencing their accessibility for outer glycan-binding proteins. ^96^

As previously elucidated, these biochemical differences among individuals in terms of SA and CD147, together with the related RBC evolutionary changes, could partly explain the reduced pathogenicity of COVID-19 in those countries where malaria and sickle cell disease are more endemic.

Furthermore, an overlap of the gene loci for the AB0 system and the loci for the iron metabolism has been elicited. ^353^ This genetic connection between cations and blood type may represent another element of discussion within the findings concerning individual genetic susceptibility.

Anyway, other authors reported no specific difference in terms of SA content in the context of the different AB0 blood types, whereas they anyway found that patients with sickle cell anemia had significantly lower SA values in comparison to the erythrocytes from healthy subjects. ^112^

In view of the wide distribution of SA molecules and of CD147 receptors on RBC, platelets and endothelium, and because of the well-known detrimental role of cation imbalance on the coagulative cascade, ^363^^,^^364^ these factors should be regarded of importance also to explain the artero-venous thromboembolic complications which may occur in COVID-19, with the formation of cation-based fibrinolysis-resistant intravascular parafibrin.

Basically, beside the traditional vision of micro-macro-thromboses based on coagulation abnormalities deriving from a number of immune-inflammatory derangements, a panoply of pro-thrombotic factors, partly based on RBC/hemoglobin dysfunction, may intervene.

Other typical clinical findings of these patients, such as hypoxemic hypoxia, pneumonia, most immune-inflammatory processes and cytokine storm/ARDS, could be equally regarded as late expressions of a multi-organ (blood, endothelium, liver and neural system included) disease, where RBC altered morphology, function and clearance, together with ferroptosis in other cell types, seem to play a major role.

SARS-CoV-2 is emerging as an easily mutating viral agent and a number of potential or documented host receptors which interact with the virus have been individualized along these two years. ^1^^,^^64^^,^^365^^,^^366^

Among these additional potential receptors, the molecules of dipeptidyl peptidase 4 (DPP4), CD209L, CD26, ciclophylin, C-lectin type receptors, toll-like receptors, neuropilin-1 and of glucose regulated protein 78 have been proposed; in fact, little if no specific data has been provided on the real role of these additional entry-ports, neither as to their possible interaction with the cation/ferroptosis pathway reported in this review.

In the latest weeks the newly emerged Omicron variant of SARS-CoV-2 has been extensively studied. A much higher diffusibility and a lower pathogenicity of this viral strain over the previous ones has been highlighted so far. ^367^

From the biophysical point of view, it has just been recorded that this variant shows a series of mutations which increase the overall positive electric charge of S protein, more specifically of S2 subunit. ^368^^,^^369^

Actually, the present review has shown how the electrical interaction between the spike protein (especially RBD of S1) and cell membrane is pivotal to determine especially pathogenicity of this viral infection. This feature derives from the formation of the new dipoles created by RBD-SA molecules, which brings the membrane electrochemical changes mentioned above, and in turn generates ion channels dysfunction.

As Omicron variant expresses a higher positive charge especially at the level of S2, it may be expected an easier cell penetration through the lipofilic fusion with cell membrane, whereas a lower ion-channeling dysfunction (putatively a lower pathogenicity) could occur.

Based on the reported evidence here, it is expected biomedical research may deepen the meaning of the interplaying pathophysiology factors elucidated in this review: cell membrane potential and ion channels, sialylated receptors, cations, RBC, hemoglobin, hepcidin. The resulting deranged ferroptosis may represent the main and ultimate cell degenerative process which characterizes the multi-organ SARS-CoV-2 attack and the final lung involvement.

## Data availability

There are no underlying data associated with this article.
