# Supplementary material for: COVID-19, Cation Dysmetabolism, Sialic Acid, CD147, ACE2, Viroporins, Hepcidin and Ferroptosis: A Possible Unifying Hypothesis
Source: F1000Res. 2022 Feb 28;11:102. Originally published 2022 Jan 27. [Version 2] doi: 10.12688/f1000research.108667.2 (PMC8921693; doi:10.12688/f1000research.108667.2)
Supplement: Peer review history

# Peer review: Reviewer response for version 2

**DOI:** 10.5256/f1000research.121457.r125616

**Published:** 2022-03-14

**Authors:** Ming Yang

**Affiliations:** 1 Department of Ophthalmology, Li Ka Shing Faculty of Medicine, The University of Hong Kong, Hong Kong, Hong Kong

The authors have addressed my questions and suggestions

Is the review written in accessible language?

Yes

Are all factual statements correct and adequately supported by citations?

Partly

Are the conclusions drawn appropriate in the context of the current research literature?

Yes

Is the topic of the review discussed comprehensively in the context of the current literature?

Yes

Reviewer Expertise:

Cell biology, COVID-19, Neural regeneration

I confirm that I have read this submission and believe that I have an appropriate level of expertise to confirm that it is of an acceptable scientific standard.

# Peer review: Reviewer response for version 1

**DOI:** 10.5256/f1000research.120075.r123133

**Published:** 2022-02-10

**Authors:** Piotr H Pawlowski

**Affiliations:** 1 Institute of Biochemistry and Biophysics, Polish Academy of Sciences, Warszawa, Poland

Based on the literature background a possible unifying hypothesis related COVID-19 to cation dysmetabolism, sialic acid, cd147, ACE2, viroporins, hepcidin, and ferroptosis were presented. It indicates three main receptors and their role in the spike docking approach and virus fusion. Further, the role of hepcidin in the accumulation of intracellular ferritin and blocking iron transport then activation of the voltage-dependent cation channels, and the action of viroporins, resulting in oscillations is discussed. The possible ferroptosis consequences were shown.

 The presented hypothesis seems to be reasonable and certainly, it has a valuable unifying role. Regarding medical practice, I think that in the future it will be necessary and interesting to relate this hypothesis to the covid cytokine storm, by a mathematical kinetic model.  As some diagnostic advantage one may consider measurements of artery-vein electromotive force oscillations in normal and covid patients.

 I am sure that this a very good paper worth to be indexed as fast as possible with no major revision.

 To make it more precise, the authors may add in Fig.1 that they mean the membrane potential as the absolute value of the difference between electric potential inside and outside the cell, but it is not necessary.

Is the review written in accessible language?

Yes

Are all factual statements correct and adequately supported by citations?

Yes

Are the conclusions drawn appropriate in the context of the current research literature?

Yes

Is the topic of the review discussed comprehensively in the context of the current literature?

Yes

Reviewer Expertise:

Biophysics, bioinformatics

I confirm that I have read this submission and believe that I have an appropriate level of expertise to confirm that it is of an acceptable scientific standard.

# Response

**Published:** 2022-02-12

**Authors:** Attilio Cavezzi

**Affiliations:** Eurocenter Venalinfa, Italy

Dear Dr. Pawlowski,

 We sincerely thank you for your comments and valuable inputs. Here below we reply (in bold character) to your suggestions and comments.

 "Based on the literature background a possible unifying hypothesis related COVID-19 to cation dysmetabolism, sialic acid, cd147, ACE2, viroporins, hepcidin, and ferroptosis were presented. It indicates three main receptors and their role in the spike docking approach and virus fusion. Further, the role of hepcidin in the accumulation of intracellular ferritin and blocking iron transport then activation of the voltage-dependent cation channels, and the action of viroporins, resulting in oscillations is discussed. The possible ferroptosis consequences were shown.

 The presented hypothesis seems to be reasonable and certainly, it has a valuable unifying role."

**Authors’ reply (AR): we appreciate your positive comments and we acknowledge that our hypothesis may represent a starting basis for future discussions about the pathophysiology of this relatively known disease.**

 "Regarding medical practice, I think that in the future it will be necessary and interesting to relate this hypothesis to the covid cytokine storm, by a mathematical kinetic model.  As some diagnostic advantage one may consider measurements of artery-vein electromotive force oscillations in normal and covid patients."

**AR: we take in due consideration your proposed topics for further studies and we will do our best to promote some additional research, about these ideas. Additionally, we have found two preliminary articles where an increased arterial stiffness has been documented in COVID-19 patients**

**Schnaubelt S, Oppenauer J, Tihanyi D, Mueller M, Maldonado-Gonzalez E, Zejnilovic S et al. Arterial stiffness in acute COVID-19 and potential associations with clinical outcome. J Intern Med. 2021 Aug;290(2):437-443. doi: 10.1111/joim.13275.**

**Jud P, Kessler HH, Brodmann M. Case Report: Changes of Vascular Reactivity and Arterial Stiffness in a Patient With Covid-19 Infection. Front Cardiovasc Med. 2021 May 12;8:671669. doi: 10.3389/fcvm.2021.671669**

**We will include and comment these works in our own manuscript.**

 "I am sure that this a very good paper worth to be indexed as fast as possible with no major revision.

 To make it more precise, the authors may add in Fig.1 that they mean the membrane potential as the absolute value of the difference between electric potential inside and outside the cell, but it is not necessary."

**AR: thanks for your appreciation. Following to the reviewer’s suggestion, we will include the proposed clarification in Fig.1 where needed.**

**We thank the reviewer for his contribution to implement and improve our manuscript**

# Peer review: Reviewer response for version 1

**DOI:** 10.5256/f1000research.120075.r121216

**Published:** 2022-01-31

**Authors:** Ming Yang

**Affiliations:** 1 Department of Ophthalmology, Li Ka Shing Faculty of Medicine, The University of Hong Kong, Hong Kong, Hong Kong

Attilio *et al.* did a short review connecting the link between COVID-19 and ferroptosis.

 In fact, the association between SARS-CoV-2 infection and Ferroptosis was firstly proposed by Yang *et al.* 2020 ^1^. Therefore, citing this paper to further support this review is essential.

 Ferroptosis was firstly proposed in 2012 and currently three pathways have been proposed: GPX-4, FSP1, and DHODH. The author should write a section summarizing recent advances about ferroptosis. It would also be useful to discuss how exactly COVID-19 links to any of them.

 It has been shown that ACE2, CD147, viroporins and hepcidin are cell membrane targets of SARS-CoV-2 spikes. On the other hand, Transferrin and ferriportin are in the charge of iron influx and efflux. How to link the two in COVID-19 patients is also worth a discussion.

 Delta and Omicron have much more spike mutations, it is important to analysis potential novel receptors other than ACE2, CD147 and viroporins that the virus may bind to.

Is the review written in accessible language?

Yes

Are all factual statements correct and adequately supported by citations?

Partly

Are the conclusions drawn appropriate in the context of the current research literature?

Yes

Is the topic of the review discussed comprehensively in the context of the current literature?

Yes

Reviewer Expertise:

Cell biology, COVID-19, Neural regeneration

I confirm that I have read this submission and believe that I have an appropriate level of expertise to confirm that it is of an acceptable scientific standard, however I have significant reservations, as outlined above.

## References

[1] : SARS-CoV-2 infection: can ferroptosis be a potential treatment target for multiple organ involvement?. *Cell Death Discov* .2020;6: 10.1038/s41420-020-00369-w 130 10.1038/s41420-020-00369-w 33251029PMC7687212

# Response

**Published:** 2022-02-12

**Authors:** Attilio Cavezzi

**Affiliations:** Eurocenter Venalinfa, Italy

Dear Dr.Yang, we sincerely thank you for your review and your inputs. Here below you find our replies in bold) to your enquiries (which are listed before each reply) and furthermore we revised our text as per your indications.

 "Attilio *et al.* did a short review connecting the link between COVID-19 and ferroptosis.

 In fact, the association between SARS-CoV-2 infection and Ferroptosis was firstly proposed by Yang *et al.* 2020. Therefore, citing this paper to further support this review is essential."

**Authors’ reply (AR): we appreciate your input. In fact, the paper you refer to was included in our manuscript at the reference number 181, as we considered this work of basic importance for our narrative review. Moreover, it was our aim to elucidate in an original manner how intracellular cation (iron namely) accumulation takes place through several biochemical mechanisms based on sialic acid molecules, CD147 and ACE2, but also based on the electrochemical changes in the cell membrane, the viroporin action and the hepcidin/ferroportin deregulated axis *etc*. Also, reference 1 of our manuscript in fact details a past publication of ours concerning ferroptosis, as we highlighted the connection between iron accumulation, ferroptosis and COVID-19 early in June 2020.**

 "Ferroptosis was firstly proposed in 2012 and currently three pathways have been proposed: GPX-4, FSP1, and DHODH. The author should write a section summarizing recent advances about ferroptosis. It would also be useful to discuss how exactly COVID-19 links to any of them."

**AR: we took note of your valuable suggestion and we included a number of lines regarding these additional pathways which pertain to ferroptosis degenerative mechanisms and their possible relationship to COVID-19.**

 "It has been shown that ACE2, CD147, viroporins and hepcidin are cell membrane targets of SARS-CoV-2 spikes. On the other hand, Transferrin and ferriportin are in the charge of iron influx and efflux. How to link the two in COVID-19 patients is also worth a discussion."

**AR: we elaborated more in depth on the relationship among hepcidin, transferrin, ferroportin and iron metabolism, including also the name “transferrin” in the title of the paragraph “f”**

 "Delta and Omicron have much more spike mutations, it is important to analysis potential novel receptors other than ACE2, CD147 and viroporins that the virus may bind to"

**AR: we took note of the suggestion and kindly remind that we highlighted the preeminent role of sialic acid molecules as receptors in the cell membrane and on the documented or potential host receptors. However we did our best to add some more information about further receptors and possible literature data connected to our hypothesis, though the evidence is not clear ye**t.

**We sincerely thank the reviewer for his help to improve the manuscript**
